# Study on the Influence of Silica Fume (SF) on the Rheology, Fluidity, Stability, Time-Varying Characteristics, and Mechanism of Cement Paste

**DOI:** 10.3390/ma15010090

**Published:** 2021-12-23

**Authors:** Hengrui Liu, Xiao Sun, Yao Wang, Xueying Lu, Hui Du, Zhenghong Tian

**Affiliations:** 1 College of Water Conservancy and Hydropower Engineering, Hohai University, Xikang Road No.1, Nanjing 210098, China; 171302020037@hhu.edu.cn (H.L.); wyao98@hhu.edu.cn (Y.W.); luxy@hhu.edu.cn (X.L.); Huid@hhu.edu.cn (H.D.); zhtian@hhu.edu.cn (Z.T.); 2 State Key Laboratory of Hydrology Water Resources and Hydraulic Engineering, Hohai University, Xikang Road No.1, Nanjing 210098, China

**Keywords:** silica fume (SF), rheology, fluidity, stability, water film thickness, micromechanism

## Abstract

In this study, the rheology, fluidity, stability, and time-varying properties of cement paste with different substitute contents of silica fume (SF) were investigated. The result showed that the effects of SF on macro-fluidity and micro-rheological properties were different under different water–cement ratios. The addition of SF increased the yield stress and plastic viscosity in the range of 2.61–18.44% and 6.66–24.66%, respectively, and reduced the flow expansion in the range of 4.15–18.91%. The effect of SF on cement paste gradually lost its regularity as the w/c ratio increased. The SF can effectively improve the stability of cement paste, and the reduction range of bleeding rate was 0.25–4.3% under different water–cement ratios. The mathematical models of rheological parameters, flow expansion, and time followed the following equations: *τ*(*t*) = *τ*_0_ + *k*_0_*t*, *η*(*t*) = *η*_0_*e^at^*, and *L*(*t*) = *L*_0_ − *k*_1_*t*, *L*(*t*) = *L*_0_ − *k*_1_*t* − *a*_1_*t*_2_. The SF slowly increased the rheological parameters in the initial time period and reduced the degree of fluidity attenuation, but the effect was significantly enhanced after entering the accelerated hydration period. The mechanism of the above results was that SF mainly affected the fluidity and rheology of the paste through the effect of water film thickness. The small density of SF particles resulted in a low sedimentation rate in the initial suspended paste, which effectively alleviated the internal particle agglomeration effect and enhanced stability. The SF had a dilution effect and nucleation effect during hydration acceleration, and the increase of hydration products effectively increased the plastic viscosity.

## 1. Introduction

During the construction of a roller-compacted concrete dam, there is a problem that some areas cannot be compacted, and the application of metamorphic concrete paste injection technology can solve this problem [1]. The stability, fluidity, rheology, and time-varying properties of metamorphic concrete paste are all important properties that need to be considered [2,3]. The stability characterizes its ability to maintain its original properties, and the bleeding rate is usually used to characterize the stability of the paste [4]. The fluidity and rheology of the paste directly influence the effect of grouting or infiltration, which in turn affects the construction quality of metamorphic concrete [5,6]. The time-varying characteristics can significantly affect the diffusion effect of paste [7,8]. However, traditional metamorphic pastes often have problems such as poor rheology, insufficient strength, and poor stability. In addition, as the main component of the paste, the extensive use of cement in grouting leads to the rapid consumption of resources and energy and the generation of harmful gases [9,10]. Therefore, considering the grouting performance, resources, environment, and cost of the metamorphic paste, it is a challenging task to improve grouting with new additives. 

Through recent research and engineering applications, some green mineral admixtures such as fly ash, basalt powder, zeolite powder, etc. have also been used in the grouting field and showed good performance [11,12,13,14,15]. However, compared with the actual demand of engineering construction, the supply of green mineral admixtures in the grouting field is still very limited. Therefore, the selection of available materials must be carefully studied to meet the requirements of environmental and economic costs while considering the good properties of metamorphic paste [16,17]. The silica fume (SF) is processed from natural quartz and fused quartz, and the production process is non-toxic, non-polluting, and low in cost. The storage of quartz is large in nature, which can meet the large demand in the short term, so it can be used as an alternative material. 

Many scholars have studied the role of SF in the performance of cement paste, mortar, and concrete and drawn corresponding conclusions. Smita Sahoo [18] conducted a comprehensive experimental study on the strength, durability, and microstructure properties of SF-containing concrete, and believed that 15% was the optimal amount of SF used in concrete. With the addition of 15% SF, the concrete’s compressive strength, sulfuric acid corrosion resistance, chloride corrosion resistance, and carbonation resistance were all improved to varying degrees. Mahdi Shariati [19] explored the effect of SF on the durability and microstructure characteristics of alkali-activated slag geopolymers and found that SF significantly increased the resistivity and reduced the water absorption rate of the paste by generating additional CSH gel. Xiaodi Dai [20] found that SF can improve the drying shrinkage and mechanical properties of alkaline activated slag cement (AASC) and has a slower structure accumulation and better workability maintenance in the early stage. Guangwei Liang [21] reported that proper SF can significantly refine the porosity of geopolymer slurry and reduce the porosity, thereby improving the mechanical properties. G. Appa Rao [22] found that the addition of silica powder increased the initial setting time of cement slurry, reduced the gas content and workability of mortar, and enhanced the early hydration reaction of C3A and C3S in paste. 

Some researchers also achieved good results by using SF in combination with other mineral admixtures or chemical additives. Aneel Kumar [23] reported that the effect of SF on the compressive strength of burnt clay bricks (BCB) was better than that of fly ash. The 4% SF increased the compressive strength of BCB by 27.55%, while the 4% FA increased the compressive strength by 17.36%. Sherong Zhang [24] combined SF with waste marble powder (WMP) and found that cellular concrete containing 10% SF and 5–20% WMP showed the best mechanical and durability properties. Mojtaba Nili [25] used concrete mixed with air and silica fume to show the best performance in salt scale tests, the addition of SF reduced the total pore volume and refined the pore distribution. The mechanism of SF in cement-based materials and concrete has also been explored. Jeong proved [26] that silica fume provides seed sites for cement hydration nucleation and subsequent acceleration and provides active silica for the pozzolanic reaction. Chenxin Ni [27] focused on the influence of silica fume fineness on the hydration process of cement paste. The hydration of cement changed with the SF particle size. Coarse DSF particles prolonged the acceleration period of cement hydration and reduced the rate of heat release of cement hydration. Coarse SF was conducive to the formation of AFm crystals. Fine SF accelerated the hydration of C3S, leading to an increase in the amount of CH. Xinming Wang [28] found that under low sulfate conditions, SF was adsorbed on the surface of hydration products as nucleation seeds, which promoted the precipitation and chemical shrinkage of hydration products. 

It can be seen from the above that SF has been widely used in the field of cement-based and concrete materials, and its influence on durability, mechanical properties, and microstructure has also been explored. However, there are few studies on the rheology and stability of silica fume in cement-based and concrete materials. These properties are important in some engineering fields. For example, in the field of grouting, rheology is a prerequisite for determining whether the grouting material is pourable. If the grouting material does not have good rheology, it cannot enter the cracks or voids to play an effective filling role. Stability determines whether the grouting material exhibits bleeding and sedimentation. Slurry plugging and poor diffusion uniformity result in poor construction quality and severe consequences. Time-varying characteristics is the scale that determines the rheological properties and stability of grouting materials with time. In addition, the current explanation of the mechanism of the influence of silica fume on the rheology and stability of cement paste is relatively simple, and new theories need to be developed to explain the influence mechanism of silica fume, which is convenient for the application of silica fume to provide theoretical guidance in the field of grouting. 

Therefore, based on the above discussion, an experimental study on the characteristics of the cement paste containing silica powder (SF) was carried out. The main purpose of this research is twofold: (i) The influence of SF on the rheology, fluidity, stability, and time-varying properties of cement paste were evaluated, which provided a theoretical basis for the application of SF in the field of grouting. (ii) The water film thickness theory, bleeding sedimentation model, microstructure observation, and hydration heat analysis methods were used to explain the mechanism by which SF changes the fresh and time-varying properties of cement paste. 

## 2. Materials and Methodology

### 2.1. Materials

Ordinary Portland cement (OPC) with a strength grade of 42.5 was used, and the physical and chemical results tested met the requirements of the Chinese standard GB175-2007. The fly ash used in the test ratio was Grade III fly ash, which conformed to the Chinese standard GB 1596-91. The loss on ignition (LOI) value of FA was 10.0% and the density was 2.1 g/cm^3^. Silicon fume was waste ash collected and processed in the process of smelting industrial silicon and ferrosilicon at high temperature by an industrial electric furnace (Beijing Basalt Stone Material Factory, Beijing, China), and it was processed by ultrafine grinding. The chemical compositions of OPC, FA, SF were analyzed by X-ray fluorescence spectrometer (XRF) (Malvern PANalytical, Shanghai, China), and the data obtained were listed in Table 1. The total contents of SiO_2_, Al_2_O_3_, and Fe_2_O_3_ in SF and FA were about 95.84% and 88.28%, respectively, higher than the minimum requirements (70%) specified in ASTM C 618, meeting the requirements for the use of mineral admixtures in cement-based materials and concrete. 

**Figure 1:**
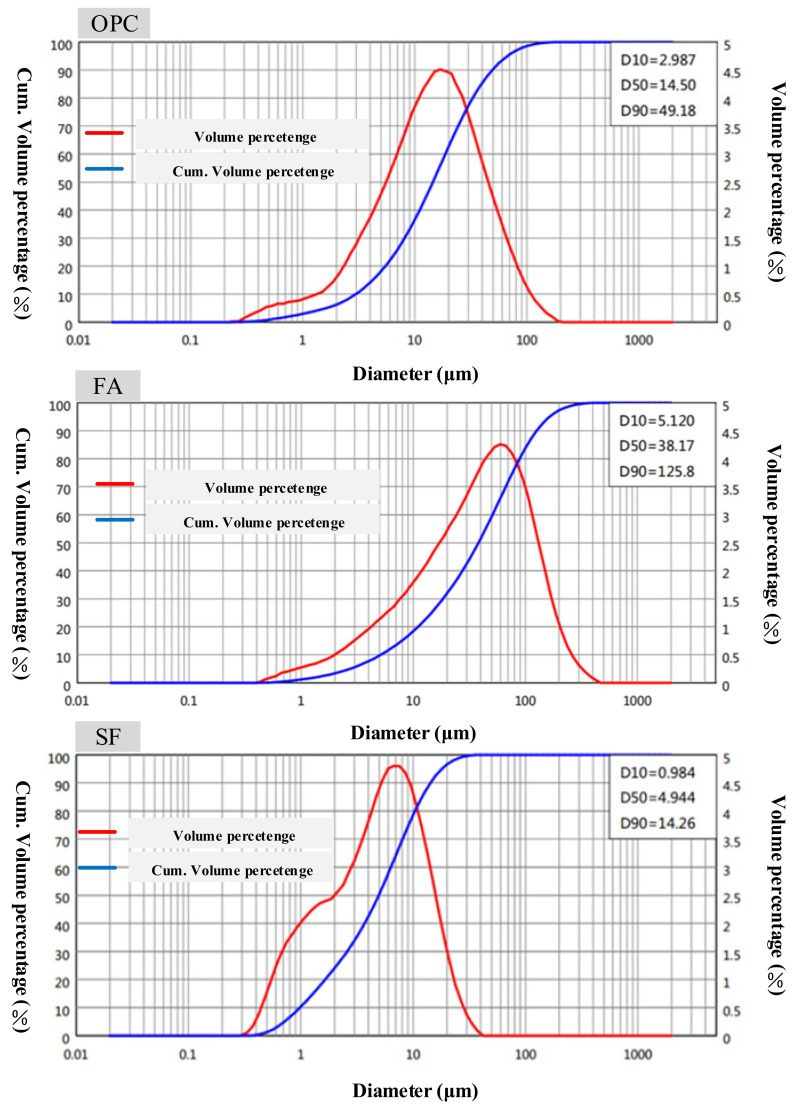
Particle size distributions of OPC, FA and SF.

The MASTERSIZER 2000 laser particle size analyzer (Malvern PANalytical, Shanghai, China) was used to analyze the particle size of OPC, FA, and SF. The particle size distribution is shown in Figure 1. The median diameters of OPC, FA, SF were 14.5 μm, 38.17 μm, and 4.94 μm, respectively. The specific surface areas of OPC, FA, and SF were 367.9 m^2^/kg, 192.0 m^2^/kg, and 866.7 m^2^/kg, respectively. The particle size of SF was much smaller than that of cement and FA, and it can effectively fill the paste.

**Figure 2:**
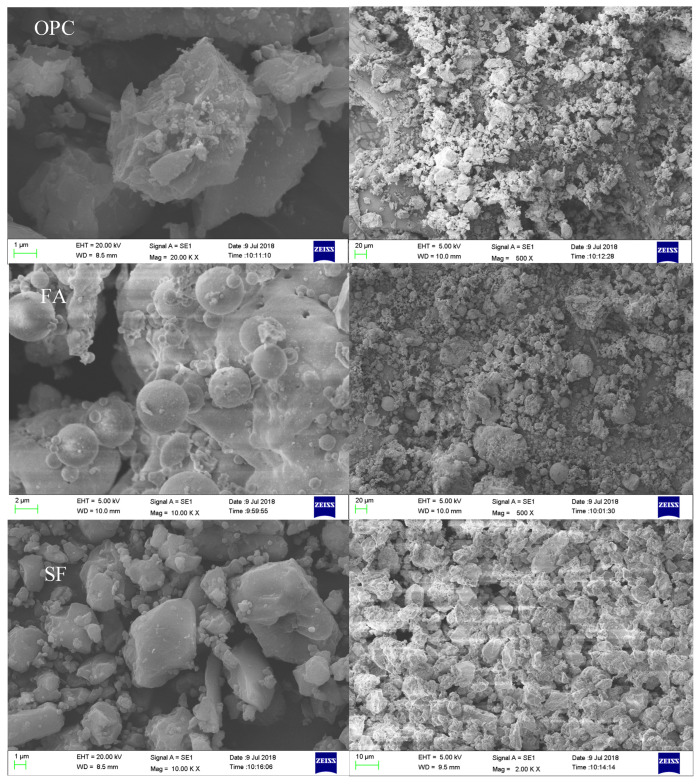
SEM morphology of OPC, FA, and SF.

The morphology of cement, fly ash, and silica fume particles was analyzed by scanning electron microscope (Carl Zeiss AG, Oberkochen, Germany), as shown in Figure 2. From the results, it can be seen that the particle size of fly ash was similar to a round shape, which can play a lubricating effect. The shape of SF was irregular prismatic, which affected the fresh properties of cement paste mainly through filling and surface effects. It can be seen from the Figure 2 that the particle size of SF was significantly smaller than FA, which conforms to the particle distribution curve shown in Table 1.

### 2.2. Experimental Program

The basis for determining the mix ratio in this study was as follows: (i) The w/c ratio of conventional metamorphic concrete paste ranged from 0.4 to 0.7, so the w/c ratios in this study were 0.4, 0.5, 0.6, and 0.7. (ii) A small amount of cement resulted in a decrease in the strength of the metamorphic concrete. Therefore, a high amount of cement was always maintained in the proportion. (iii) Due to the needs of engineering economic costs, the total substitute content of mineral admixtures in this study was about 50%. The proportion of all cement samples maintained that the cement content was 50% of the total cementing material, while SF and FA were 50% together, with SF increasing from 0 to 50% in increments of 10%. In summary, the test blending ratio of paste is shown in Table 2. The paste was stirred by the NJ-160 type pure paste mixer (Tianjin Qingda Test Instrument Manufacturing Company, Tianjin, China) in accordance with the JC/T729 specification. It rotates slowly at 140 r/min for 120 s, stops mixing for 15 s, and then rotates quickly at 285 r/min for 120 s. All cement paste samples were prepared and tested in an underground laboratory with a temperature controlled at 22 ± 2 °C and a humidity of 90%.

The rheological curve of the paste was measured by a rotary rheometer (Shanghai Precision Instrument Company, Shanghai, China), and the plastic viscosity and yield stress were obtained. The results of the flow spread test were used to characterize the fluidity of the paste. The bleeding rate of the paste based on different mix ratios and moments was used to evaluate the stability change. Based on the data statistical analysis method, the rheological parameters and fluidity of the paste were tested and determined, and the time-varying curve equations were obtained.

The packing density and void ratio were measured by wet filling method; the water film thickness of the particles was calculated based on the volume of w/c ratio, packing density, and void ratio; and the relationships between water film thickness and flow and rheological parameters were discussed based on correlation analysis. Based on the Stokes formula, paste density, viscosity, particle radius, particle density, and particle shape coefficient were used to estimate the sedimentation rate of particles. An upright fluorescence microscope (Shenzhen Boshida Optical Instrument Comapny, Shenzhen, China) was used to observe the agglomeration of particles and changes in flocculation structure at different times. The iButton temperature recorder (Malvern PANalytical, Shanghai, China) was used to measure the heat of hydration temperature and the heat of hydration curve was drawn. 

### 2.3. Test Methods

#### 2.3.1. Flow Spread

Following the method adopted by Hajime Okamura and Masahiro Ouchi [29], a mini slump cone was used to measure flow spread. When testing the time-varying fluidity, it was measured every 20 min within 300 min, and every 10 min thereafter. 

#### 2.3.2. Rheological Properties

The yield stress and plastic viscosity of the paste were tested with an NXS-11A rotary viscometer (Shanghai Precision Instrument Company, Shanghai, China). Specific steps were as follows: (a) After adjusting the level of the base, an appropriate amount of paste was put into the outer cylinder. (b) The central axis of the measuring inner cylinder was connected to the measuring head and fixed with screws. (c) The jacket was aligned with the paste and inserted into the inner cylinder, and rotated and fixed. (d) The knob was turned on the probe to “work”, then the speed shift was gradually turned from 1 to 15, while reading the scale and recording the measured value.

The yield stress and apparent viscosity can be obtained from the following equation:

(1) $$\tau =\tau_{0}+\mu \times \gamma +c\times \gamma^{2}$$

where *τ* and *τ*_0_ are shear stress and yield stress; *γ* is the shear rate; *μ* is the plastic viscosity, and *c* is the second-order parameter. Finally, the best fitting curve was obtained using the regression analysis, and the corresponding parameters *τ*_0_ and *μ* were determined as the yield stress and apparent viscosity of the cement paste samples.

#### 2.3.3. Bleeding Rate

The measurement of bleeding rate conformed to the Chinese standard specification JT/T 946-2014. A 120 mm height plexiglass container was filled with cement paste to about 100 mm height, the cap of the plexiglass was sealed to prevent the moisture change. The initial surface of the cement paste was denoted as *d*_0_. The bleeding layer volume *d*_1_ was measured every 10 min (up to 60 min). The bleeding rate was calculated by dividing the height of the bleeding water with the height of the original paste, as shown below:

(2) σ=(d1−d0)100

#### 2.3.4. Observation of Microstructure

The flocculation of the paste can be observed under the optical microscope to absorb and disperse [30]. According to the flocculation and dispersion inside the paste, the property change of the paste can be judged. The microscope used for the experimental observation was an upright fluorescent microscope, and a plastic dropper, a glass slide, a cover glass, and a 20 mL test tube needed to be prepared. The specific steps were as follows: the 1 mL of prepared cement paste was sucked with a glue-tip dropper, and then 19 mL of clean water was slowly added to dilute it by 20 times. Then, another dropper was used to pipette the solution evenly onto the glass slide, and the cover glass was gently placed. When the microscope was switched to magnify and observe, after adjustment, it was found that the observation was more suitable when 400 times.

#### 2.3.5. Heat of Hydration

The measurement of heat of hydration conformed to the Chinese standard specification ASTM C1702-2017. A measure of 500 g cement paste was weighed and put into the thermal insulation box (Malvern PANalytical, Shanghai, China), and then the thermal insulation box was placed in a constant temperature and humidity environment. The time interval of the hydration heat temperature was set and measured in the iButton temperature recorder (this test was recorded every 3 min), and the temperature unit was adjusted to degrees Celsius. After the iButton temperature recorder started working, the process of cement hydration heat release was automatically measured. In this experiment, the hydration heat release process of the paste with different mixing ratios of w/c of 0.4, 0.5, and 0.6 was tested. 

#### 2.3.6. Packing Density and Water Film Thickness

The measurement of the packing density of mixed powders used the wet filling method [31,32]. The principle of the wet filling method was that the solid concentration of the paste changed with the w/c ratio. When the w/c ratio was large, the solid particles were dispersed in the water, and the increase in the w/c ratio resulted in a decrease in the solid concentration. When the w/c ratio was small, the amount of water was not enough to form a paste with the solid particles, resulting in a decrease in the solid concentration as the w/c ratio decreased. Therefore, there was an optimal w/c ratio to maximize the solids concentration of the paste. At this time, the maximum solid concentration that occurred when the particles were tightly packed with each other was considered to be the packing density of the mixture. With the packing density and specific surface area of each cementitious system, the WFT can be obtained from the following equations:

(3) $$u_{0}=\mu_{w}-\mu_{s}\times \frac{\left(1-\mu_{pd}\right)}{\mu_{pd}}$$

(4) $$A=A_{0}\times R_{0}+A_{Z}\times R_{Z}+A_{B}\times R_{B}$$

(5) $$WFT=\frac{u_{0}}{A}$$

where *μ_w_* is the volume of mixing water; *μ_s_* is the volume of solid particles; *μ_pd_* is the packing density of particles; *A*_0_, *A_Z_*, and *A_B_* are the specific surface areas of OPC, ZP, and BP, respectively; *R*_0_, *R_Z_*, and *R_B_* are the volumetric ratios of OPC, ZP, and BP to the total solid volume, respectively.

## 3. Results and Discussion

### 3.1. Fluidity

**Figure 3:**
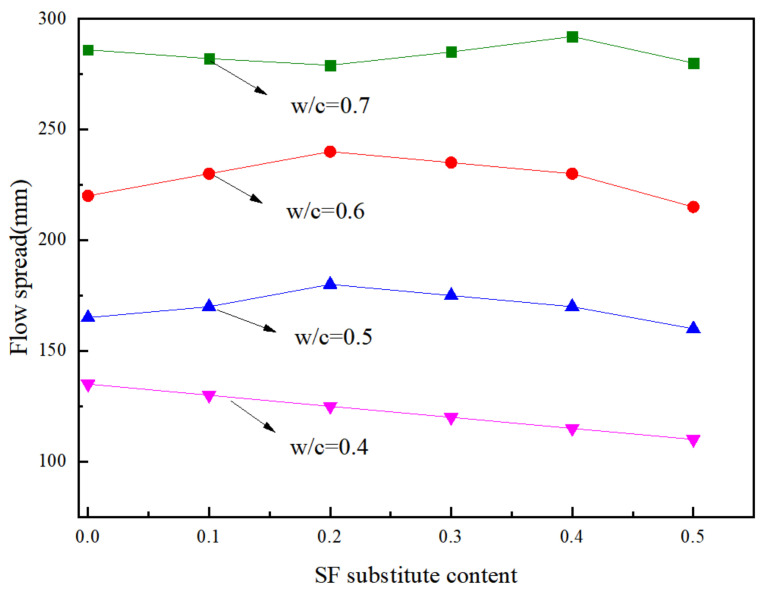
Variations of flow spread with w/c at different SF substitute contents.

The flow spread results are plotted against SF contents for different w/c ratios in Figure 3. When the w/c ratio was 0.4, the fluidity order of the paste was: A1 > A2 > A3 > A4 > A5 > A6; When the w/c ratio was 0.5 or 0.6, the fluidity of the paste was A3 > A2 > A1 > A4 > A5 > A6. While there was no direct relationship between the fluidity and the substitute contents of SF at a w/c ratio equal to 0.7. The results showed that the changing regularity with the increasing substitution of SF under different w/c ratios was not the same. The addition of SF reduced the fluidity of the paste when the w/c ratio was 0.4. The fluidity showed a trend of first increasing and then decreasing with the addition of SF when the w/c ratio was 0.5, 0.6. When the w/c ratio was 0.7, the fluidity of the paste was sufficiently excellent, and the SF no longer had the promoting effect [33]. 

### 3.2. Rheological Properties

**Figure 4:**
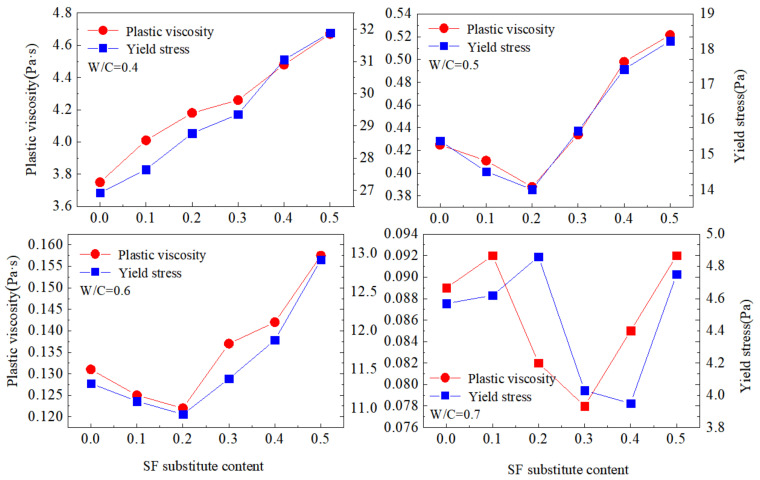
Variations of yield stress and apparent viscosity with w/c at different SF substitute contents.

Yield stress is an index that characterizes the strength of the colloid network within the paste. It was greatly affected by the strength of the attractive force between the particles, which characterized the difficulty of the paste starting to flow [34]. The plastic viscosity was the degree of difficulty for the internal structure of the paste to hinder the flow [35]. The yield stress and apparent viscosity results are plotted against SF contents for different w/c ratios in Figure 4. 

Under different substitution of SF and w/c ratio, the changes in the yield stress and plastic viscosity of the paste were different. The yield stress and plastic viscosity of the paste increased with the increase of SF substitute contents at a w/c ratio equal to 0.4. When the w/c ratio was 0.5 or 0.6, the addition of SF caused the rheological parameters of the paste to decrease first and then increase. The addition of SF did not show obvious regularity to the plastic viscosity and yield stress of the paste when the w/c ratio was 0.7.

The SF mainly changed the rheology of the paste through filling and surface effects. The particle size of SF was significantly smaller than that of fly ash and cement. The addition of SF played an effective filling role and optimized the gradation of the mixed powder, which released the free water of the flocculation structure to reduce the friction between the particles, so the incorporation of SF can reduce the yield stress and plastic viscosity of the paste. In addition, the specific surface area of SF particles was large, and the paste required more free water to cover the surface of the particles, and the water content demand of the paste increased. Therefore, the incorporation of SF can increase the rheological parameters of the cement paste. The increase or decrease in rheological parameters depended on the net effect of filling and surface effect [34].

### 3.3. Bleeding Rate

The stability represents the ability of the cement paste to resist particle sedimentation and free water precipitation under the effect of gravity. Generally, the bleeding rate of the paste during a specific period of time is used to evaluate the stability. The traditional metamorphic paste had the characteristics of high w/c ratio, so the bleeding and sedimentation of the paste during transportation and grouting were more serious. Therefore, the effect of different substitute content of SF on the improvement of stability was studied. 

**Figure 5:**
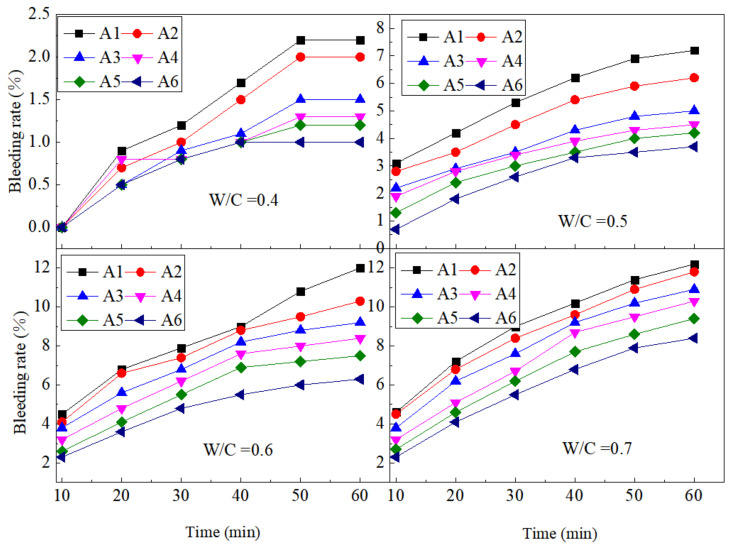
Variations of bleeding rate with w/c at different SF substitute contents.

The bleeding rate results are plotted against SF contents for different w/c ratios in Figure 5. Under different w/c ratios, the bleeding rate of the paste decreased with the increase of SF. At the time of the first measurement for 10 min, the greater the w/c ratio, the more obvious the improvement effect of SF on the bleeding rate. The A6 paste was reduced by 0%, 0.6%, 1.2%, and 1.8% compared to the control paste A1 when the water-binder ratio was 0.4–0.7. The bleeding rate was mainly related to the particles, flocculation structure and free water content of the paste at different times. The radius of the SF particles was smaller than that of cement and fly ash particles, so its sedimentation rate under the effect of gravity was also relatively small. At the same time, the large specific surface area of the SF particles led to an increase in the amount of free water adsorbed, which reduced the upward flow of free water. Therefore, the incorporation of SF can effectively reduce the bleeding rate of cement paste [36]. 

The bleeding rate of cement pastes with different w/c ratios increased over time, and the incorporation of SF can reduce the tendency of the bleeding rate to increase with time. Taking the w/c ratio as 0.6 as an example, the bleeding rate of A6 paste in 20–60 min increased by 1.8%, 1.6%, 1.1%, 0.8%, and 0.6% compared to the initial measurement at 10 min, while the control paste A1 increased by 1.9%, 1.6%, 1.2%, 1.1%, and 0.6%. Cement paste was a constantly changing system, and the internal hydration and particle movement were constantly changing. The particles continue to settle, and the free water moved upward until a balanced system was formed. This was also the reason why the bleeding rate of the paste continues to increase over time. As time increased, the sedimentation rate of the particles became smaller, and the paste gradually reached a stable state. Therefore, although the bleeding rate continues to increase over time, the increase degree decreased. Since the sedimentation rate of SF particles was lower than that of fly ash particles, the increase in bleeding rate during the sedimentation process also decreased with the addition of SF [37].

### 3.4. Time-Varying Properties

#### 3.4.1. The Regularity of Yield Stress with Time

**Figure 6:**
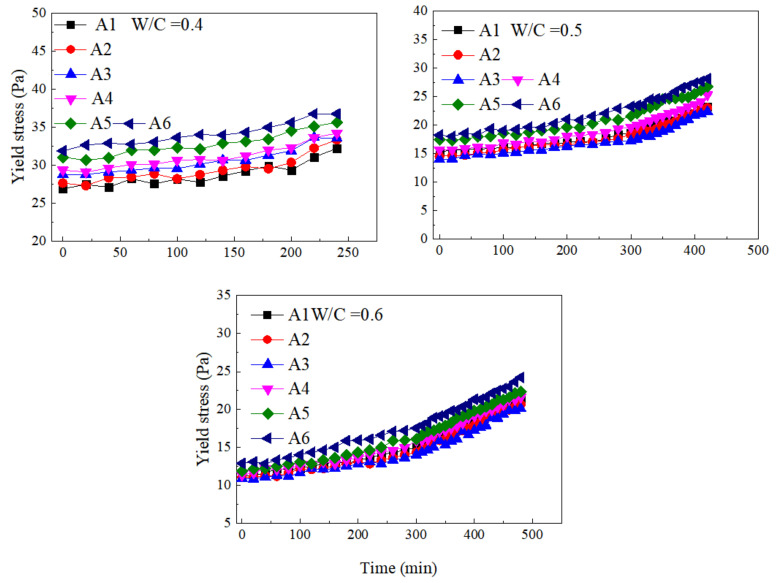
Variations of yield stress at different w/c ratios and SF substitute contents with time.

Figure 6 presents the variations of yield stress at different w/c ratios and SF substitute contents with the increase of time. The yield stress of paste containing SF showed a slow increase trend under different w/c ratios. According to the curve change trend, it can be divided into two change time periods: the first period was from 0 to 300 min, and the second period was after 300 min. The increase in the yield stress of the first period was small. The increase in yield stress at this period was mainly due to the formation of early hydration products, but the production of a small amount of hydration products did not significantly affect the yield stress of cement paste. The yield stress of the paste in the second period still showed an increasing trend, and the increase was greater than in the first period. The hydration reaction entered an accelerated period in the second period, and the hydration products CH and C-S-H were continuously generated. The dense network structure formed inside the slurry increased the yield stress of the cement paste. 

In the first stage, the incorporation of SF did not notably increase the yield stress with time. At this period, the contribution of FA and SF to the hydration reaction was similar. Therefore, SF did not significantly increase the degree of hydration in the first period, while the incorporation of SF in the second period can significantly affect the yield stress of the paste. The hydration reaction in the paste entered the accelerated period, and the particle size of silicon powder was small, which could improve the nucleation site of hydration products and contribute to the hydration reaction. Moreover, the nucleation effect and filling effect provided by SF were stronger than FA.

In addition, in the research of metamorphic concrete grouting technology, in order to more accurately predict the spread of the grout in the roller-compacted concrete, the equations of the yield stress of the cement paste mixed with SF over time were obtained, as described in Table 3. The regression coefficients R^2^ of the above-mentioned fitting equations all exceeded 0.95, so it can be summarized in the form of *τ* = *τ*_0_ + *k*_0_*t* and *τ* = *τ*_1_ + *k*_1_*t*, where *k*_0_ and *k*_1_ were parameters related to the amount of SF and the w/c ratio, and *t* was the time.

#### 3.4.2. The Regularity of Plastic Viscosity with Time

**Figure 7:**
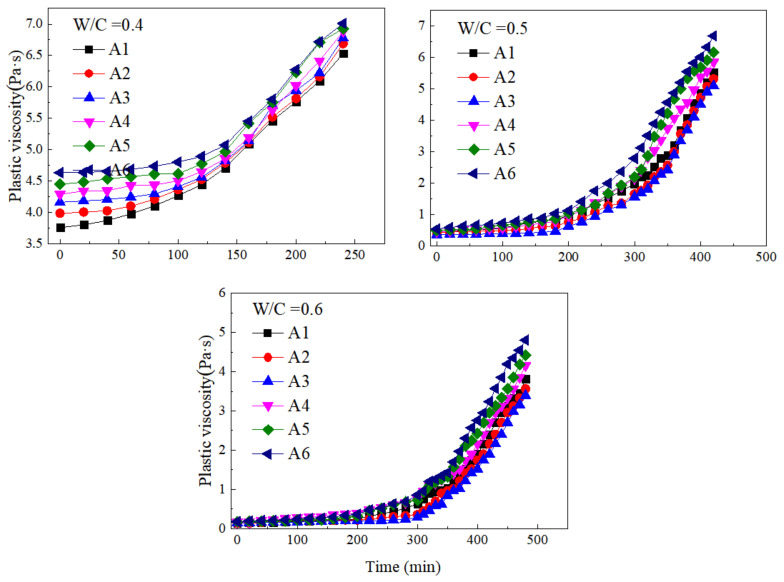
Variations of plastic viscosity at different w/c ratios and SF substitute contents with time.

Figure 7 presents the variations of plastic viscosity at different w/c ratios and SF substitute contents with the increase of time. The plastic viscosity of paste containing SF showed an exponential growth trend under different w/c ratios. In the early period of induction, the increase in the plastic viscosity of the paste was small. At this time, the main source of plastic viscosity increase was the formation of hydration products in the early period of induction. The hydration products increased the energy consumption during paste flow, but the low production of hydration products within the paste in the early period made the plastic viscosity increase slowly over time. During the accelerated period of hydration, the plastic viscosity was in a state of rapid growth, and the curve showed an exponential growth trend. A large amount of hydration products formed a flocculation structure inside the paste, and part of the free water was consumed in the hydration reaction and converted into flocculation water and bound water. The ability of the internal structure of the paste to hinder the flow of the slurry had increased, which made the plastic viscosity of the paste increase over time. 

In the early and middle of the induction period, the ability of FA and SF to promote hydration was limited, and the formation of flocculated structures within the paste was small. Therefore, SF did not significantly increase the plastic viscosity of the paste in the early and middle of the induction period, while in the accelerated hydration period, the role of SF could be effectively brought into play. The nucleation and filling of SF promoted the hydration reaction and significantly increased the plastic viscosity of the paste. 

The equations of the plastic viscosity of the cement paste containing SF over time were obtained, as described in Table 4. The results showed that the regression coefficient R^2^ of the curve fitting using the exponential function was all above 0.9, so it can be considered that the fitting effect was good. The form of the fitting equation was *η*(*t*) = *η*_0_*e^bt^*, where *b* is a parameter related to the amount of SF and the w/c ratio, and *t* is the time. 

#### 3.4.3. The Regularity of Flow Spread with Time

**Figure 8:**
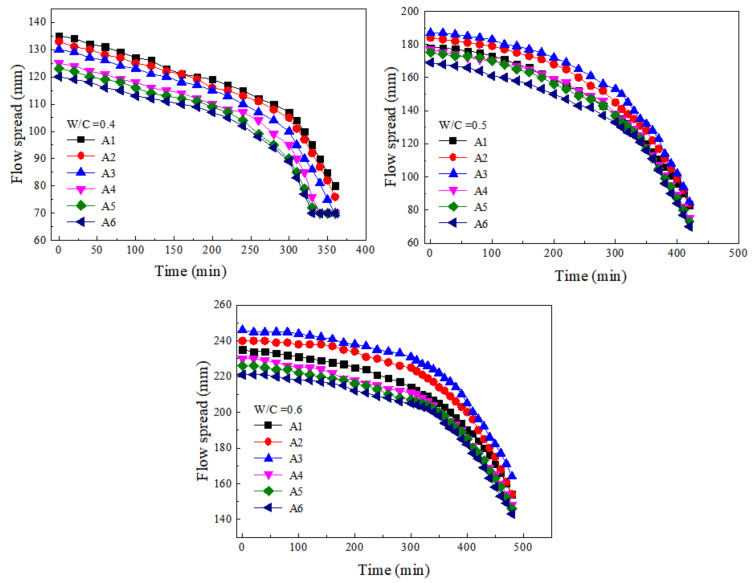
Variations of flow spread at different w/c ratios and SF substitute contents with time.

Figure 8 presents the variations of flow spread at different w/c ratios and SF substitute contents with the increase of time. The flow spread in the early stage of induction period was slowly attenuated, showing a linear decrease trend; at this time the paste still had good fluidity. The flow spread attenuation in the middle of the induction period was still not large, and the metamorphic concrete paste at this period was still workable. After entering the hydration acceleration period, the flow spread loss rate of the paste began to increase, and the flow spread–time curve showed an increasing slope. At this period, the fluidity and the permeability of the paste decreased and the rheological parameters increased. The change of fluidity was not conducive to the spread of grout in roller compacted concrete, thus affecting the construction quality. The reason for the influence of SF on the fluidity with time was similar to the explanation of yield stress and plastic viscosity. 

**Figure 9:**
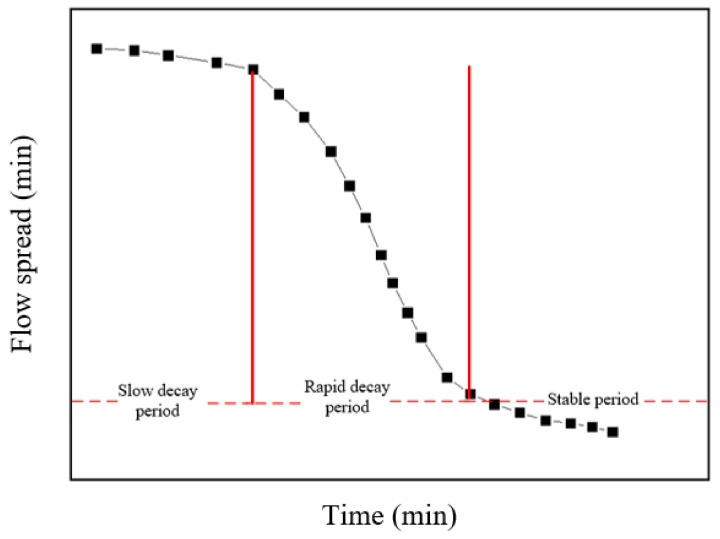
Trend of fluidity of cement paste containing SF over time.

The trend of fluidity of cement paste containing SF over time is shown in Figure 9. During the slow decay period of the fluidity of the paste containing SF, the paste had good diffusion performance and was suitable for construction. During the rapid decay period of the fluidity of the paste containing SF, the paste diffusion performance decreases quickly, and the grouting needed to be completed as soon as possible at this period. The paste gradually tended to the stable period and no longer had fluidity; its fluidity gradually stabilized and was not constructable [38]. 

The fluidity–time curve of cement paste containing SF was suitable for fitting in sections, that is, the slow decay period and the rapid decline period needed to be fitted separately. The curve was flat during the slow decay period, and the curve was fitted with a linear relationship; during the rapid decay period, the curve could be fitted with a quadratic polynomial. The equations of the flow spread of the cement paste containing SF over time were obtained, as described in Table 5. The form of the fitting equation was *L*(*t*) = *L*_0_ − *k_t_* and *L*(*t*) = *L*_1_ + *b*_0_*t* − *a*_1_*t*^2^. Where *L*_0_ was the initial fluidity of the paste, and *k*, *b*_0_, and *a*_1_ were parameters related to the w/c ratio and the substitute content of SF. The equation obtained by fitting can reflect the change of the fluidity of the cement paste over time, which provided a reference for the grouting application of metamorphic concrete paste. 

## 4. Mechanism Analysis

### 4.1. Water Film Thickness (WFT)

**Figure 10:**
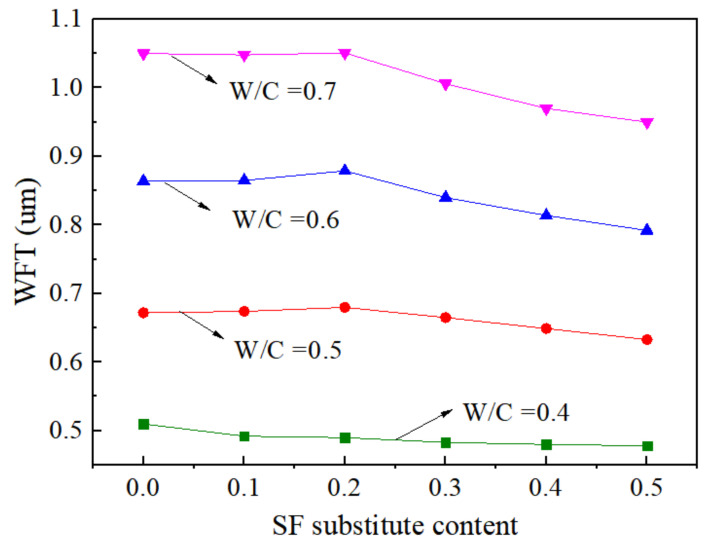
Variations of WFT with w/c at different SF substitute contents.

Figure 10 presents the variations of WFT at different w/c ratios and SF substitute contents. The SF played different roles under various w/c ratios. When the w/c ratio was 0.4, the increase substitute content of SF reduced the WFT, while the WFT increased first and then decreased with the increasing substitute content of SF at a w/c ratio equal to 0.5, 0.6, and 0.7. When the w/c ratio was at a low level, the content of water used to fill voids and flow in the paste was small. The addition of SF increased the specific surface area of the paste, and the WFT showed a trend of decreasing. When the w/c ratio gradually increased, the WFT first increased and then decreased due to two reasons. The SF increased the packing density of the mixture and released free water to increase the lubricating effect of the paste. In addition, the specific surface area of SF was significantly higher than that of cement and FA, so the addition of SF would aggravate the total specific surface area of mixture. The WFT was increased or decreased depending on the net effect of filling and surface effects. For samples A1 to A3, the net effect of the filling and surface effects of SF was positive, and the WFT increased, while samples A4 to A6 increased the packing density, but their specific surface area had a greater negative effect on fluidity. The WFT was reduced, reducing the fluidity of the cement paste.

**Figure 11:**
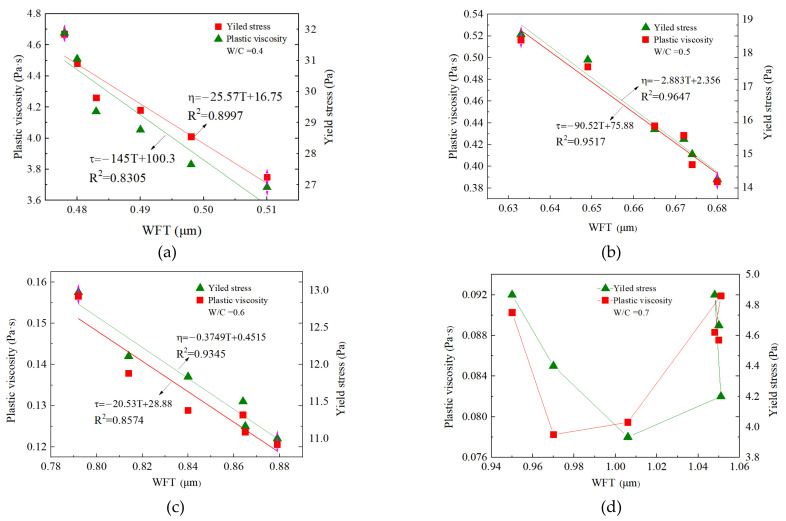
WFT vs. yield stress and plastic viscosity. (**a**) w/c = 0.4; (**b**) w/c = 0.5; (**c**) w/c = 0.6 and (**d**) w/c = 0.7.

The yield stress and apparent viscosity is plotted in Figure 11 as a function of WFT for SF contents at different w/c ratios. There was a good linear relationship between the WFT and the plastic viscosity and yield stress of cement paste when the w/c ratio was 0.4, 0.5, and 0.6. The correlation coefficient R^2^ of the fitting curve was higher, indicating that the WFT was an important factor determining the yield stress and plastic viscosity. While there was no correlation between WFT and rheological parameters when the w/c ratio was 0.7, the change in the WFT at this time was not the cause of the change in the yield stress and plastic viscosity. Therefore, only when the w/c ratio was within a certain range, the WFT was an important factor in controlling rheological parameters.

**Figure 12:**
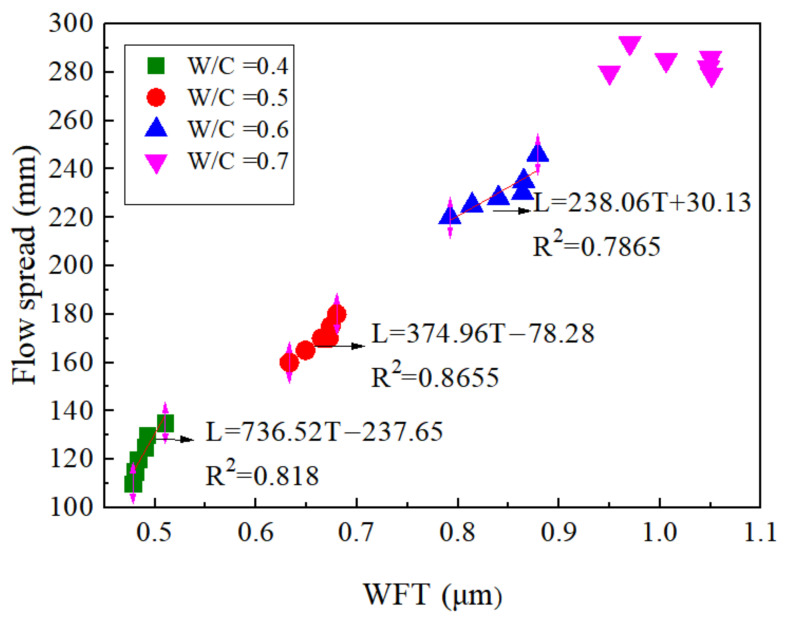
WFT vs. flow spread.

The flow spread is plotted in Figure 12 as a function of WFT for SF contents at different w/c ratios. Similar to the conclusion of rheological parameters, the flow spread was also well correlated with the WFT when the w/c ratio was 0.4, 0.5, and 0.6, while there was no good correlation between the WFT and flow spread at a w/c equal to 0.7. 

According to the above analysis, there was a direct relationship between the WFT and the flow parameter and rheological parameters of the cement paste. The WFT was an important factor in determining the rheology and fluidity of the paste, but there was an upper limit for the applicable w/c ratio. The rheology and fluidity of cement paste were mainly controlled by three forces: colloidal attraction force, gravity, and Brownian force [39]. The WFT can characterize the average distance between the interacting particles within the paste, and the colloidal attraction force and Brownian force were both related to the particle spacing. There was a threshold value of WFT to affect the fluidity and rheology of cement paste [40]. When WFT exceeded the threshold value, the change of WFT had little influence on Brownian force and colloid attraction force, and the addition of mineral admixtures had little influence on flow and rheological parameters. In contrast, when WFT was lower than its threshold value, the same amount of WFT changes had greater influence on Brownian force and colloid attraction force, and the additional mineral admixtures had significant influence on flow and rheological parameters. 

Corresponding to the above phenomenon, the addition of SF can effectively change the WFT at a low w/c ratio, thereby changing the Brown force and colloidal attraction force to affect the fluidity and rheology of the cement paste. In contrast, the addition of SF had little effect on Brownian force and colloidal attraction force under high w/c ratio. At this time, the change of WFT did not significantly change the flow parameters and rheological parameters of the cement paste. 

### 4.2. Bleeding Sedimentation Model

**Figure 13:**
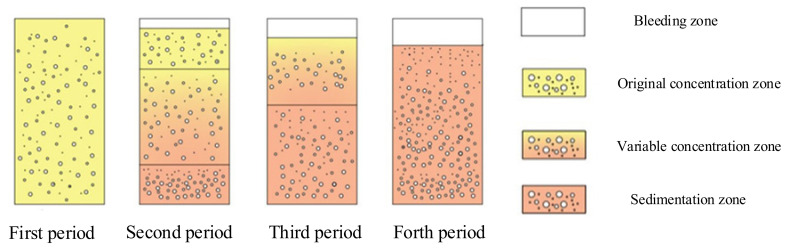
Schematic diagram of bleeding settlement model.

The bleeding sedimentation model was established to explain the phenomenon that the bleeding rate of cement paste containing SF changes with time. It was assumed that the prepared cement paste was an isotropically homogeneous suspension. Under the effect of gravity, the particles of cement, FA, and SF began to sink, and water began to flow upward. According to the movement of particles and the performance of each part of the paste, the sedimentation and bleeding process can be divided into four time periods and four zones of paste, as shown in Figure 13. 

The four different zones in the suspension were: bleeding zone, original concentration zone, variable concentration zone, and sedimentation zone [40]. The bleeding zone was mainly free water, and there may also be a small number of tiny particles. The original concentration zone was a uniformly dispersed zone where the paste maintains its original properties. The variable concentration zone was the zone where the properties were constantly changing during the particle sedimentation process. The sedimentation zone was the zone where the particles were already stable and deposited. In the first period, the slurry was able to maintain the complete dispersion of the components and maintain a relatively stable state. In the second period, the gravity of the particles within the paste dominated the colloidal attraction force, and the particles began to settle, while the water flowed upward. The paste was not a completely homogeneous suspension at this period. Except for the original concentration zone, the bleeding zone, the variable concentration zone, and the sedimentation zone existed at the same time. Since the sedimentation rate of particles was not the same and the particles were not stationary, the solid concentration in the variable concentration zone was constantly changing. In the third period, the particles continued to settle, the original concentration zone disappeared, and the suspension consisted of the other three zones. In the fourth period, particle sedimentation was basically complete, only the bleeding zone and the sedimentation zone were present. 

The reason why SF changed the bleeding rate was explained by calculating the particle sedimentation rate using Stokes formula [41].

(6) $$\nu =\frac{kr^{2}(\rho -\rho_{0})g}{\mu }$$

where: *ν* is the sedimentation rate of the particles, cm/s; *k* is the particle shape coefficient; *r* is the particle radius, cm; *ρ* is the particle density, g/cm^3^; *ρ*_0_ is the density of the dispersion medium, g/cm^3^; *g* is the acceleration due to gravity, M/s^2^; and *μ* is the viscosity of the dispersion medium, Pa·S.

The description of the particle shape factor was based on its own physical size parameters. The study of the shape factor of microsilica particles on a three-dimensional scale was too complicated and was not the main purpose of this research. Therefore, the shape factor of microsilica particles on a two-dimensional scale was used to simplify the study. The description method adopted was to modify the Blaschke coefficient to determine the two-dimensional shape of the silicon powder particles, and the calculation formula was as follows [42]:

(7) $$C_{b}=\frac{2\sqrt{\pi A}}{P}$$

where *A* is the projected area of the two-dimensional particle and *P* is the perimeter of the projected two-dimensional particle. 

**Figure 14:**
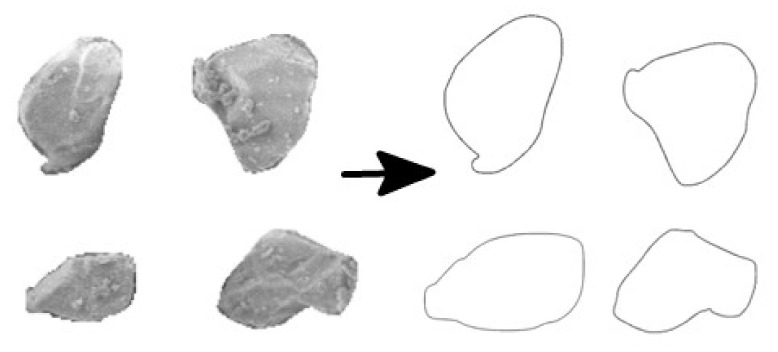
Schematic diagram of two-dimensional image processing of SF in two-dimensional scale.

Since fly ash was spherical particles, the value of the modified Blaschke coefficient of fly ash is 1. It was only necessary to obtain the value of silica powder from the derivation formula of the modified Blaschke coefficient. According to the two-dimensional image of the SF particles, the basic parameters of the perimeter and the projected area of the particles can be obtained, as shown in Figure 14. 

**Figure 15:**
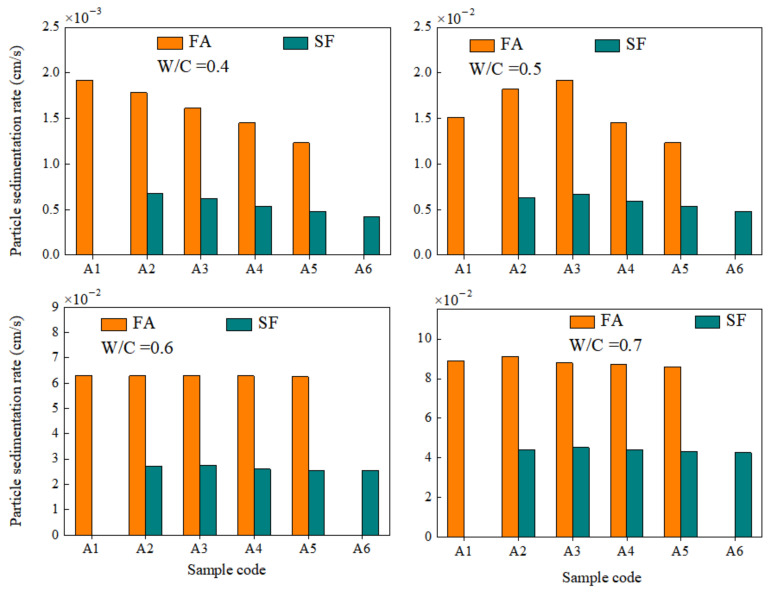
The sedimentation rate of SF and FA particles with different w/c ratios.

The particle shape coefficient of this independent sample can be estimated as a whole of SF particles. The median particle shape coefficient of this sample was 0.88. Therefore, the shape coefficient of the SF particles in the paste was determined to be 0.88 and incorporated into the calculation formula. In estimating the particle sedimentation rate, in order to reduce the amount of calculation, the parts with relatively concentrated particle size distribution in the SF and FA were respectively substituted. According to Figure 1, it can be seen that the distribution of fly ash at D_50_ = 14.94 was relatively concentrated, while the SF was concentratedly distributed at D_50_ = 4.94. The sedimentation rate results of the particles are shown in Figure 15.

The results showed that the sedimentation rate of the paste particles increased with the increase of w/c ratio. The increase in the w/c ratio reduced the density of the paste, and the decrease in the plastic viscosity resulted in an increase in the sedimentation rate of the particles, so the increase in the w/c ratio increased the bleeding rate of the paste. Under the conditions of the same w/c ratio and mixed ratio, the sedimentation rate of SF was lower than that of FA. Although the density of SF was slightly higher than that of FA, the content of fine particles in SF was higher than that of FA. Therefore, as the substitute contents of SF increased, the bleeding rate of the paste continued to decrease. 

### 4.3. Microstructure

**Figure 16:**
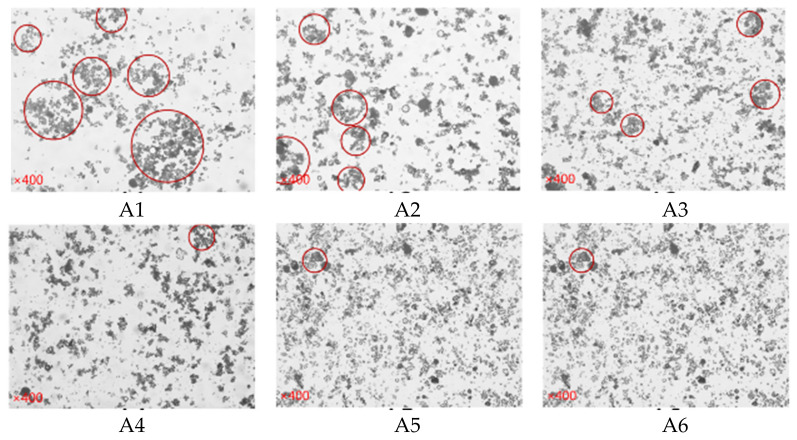
Microstructure of the cement paste containing SF.

The dispersion of SF particles was one of the key factors affecting the properties of cement paste. The excellent dispersibility of SF can also be directly verified by observing the microstructure of the paste. Figure 16 shows the microstructure effect of the cement paste under the microscope when the paste is formed for 60 min. In the figure, the highlighted part shows a more flocculated structure. The agglomeration between particles in the A1 sample was more serious, and the size of the flocculation structure was too large. After the A2 sample was mixed with SF, although the flocculation structure still existed in the paste, the particle agglomeration situation was different from that of the A1 sample. The agglomeration of fly ash cement particles was improved, and the size of the flocculation structure was reduced. The number of flocculation structures with larger sizes of A3~A6 decreased, and the distribution of flocculation structures was not too concentrated. 

There were two reasons for this phenomenon: (i) The particle size of SF was smaller than the particle size of fly ash and cement. After the silica powder was mixed, the particles were filled between the cement and FA particles. The increase of the particles spacing made it difficult to agglomerate, which played a physical role in dispersion and dilution. (ii) The incorporation of SF increased the Zeta potential and electrostatic repulsion of the paste, making it difficult for the particles to adsorb, agglomerate and flocculate. Therefore, under the combined effect of the dispersion and dilution of SF particles and the increase in the Zeta potential of the paste, the agglomeration of the particles within the paste and the excessive large-size flocculation structure had been effectively alleviated. 

### 4.4. Heat of Hydration

**Figure 17:**
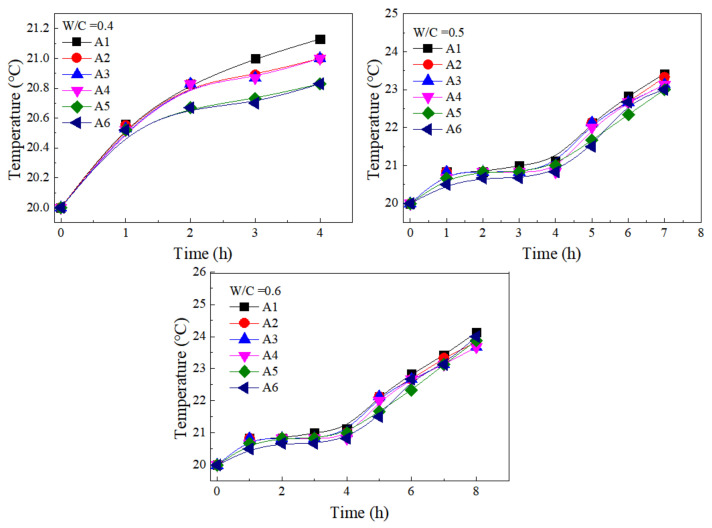
Heat of hydration of cement paste containing SF under different w/c ratios.

Figure 17 presents the heat of hydration of cement paste containing SF under different w/c ratios. The temperature rise of the paste under different w/c ratios and SF substitute contents was consistent with increasing time. The heat of hydration rose sharply within 1 h, then the temperature fluctuated low between 0 and 1 h, and then began to rise sharply after 5 h. In the first period, 0–1 h, the heat of hydration was mainly derived from the dissolution of cement, which reacted with hydration to produce hydration products such as AFt. The lack of active substances in SF and FA cannot directly participate in the hydration reaction. Therefore, the flow and rheological parameters of the cement paste mixed with SF in the first period changed little. In the second period, 1–5 h, the fluidity and rheology change range of the paste containing SF was about the same as that of the control paste. The reason for the slow hydration reaction was the slow dissolution of minerals. Although FA and SF had the effect of promoting hydration, they did not play a role in this period. During the acceleration period of the third stage of hydration reaction (after 5 h), there was a large amount of C-S-H growth during this period. According to the theory of crystal growth, the rate and amount of C-S-H generation depended on the number of crystal nuclei. The increase of nucleation sites inside the paste increased the rate of C-S-H formation and produced more hydration products. Both FA and SF had heterogenic nucleation and could provide nucleation sites for the growth of C-S-H, but SF has a larger specific surface area, which can provide more nucleation sites. Moreover, SF had a stronger filling effect than FA due to its small size, which can play a filling role and provide more free water. Therefore, the contribution of SF to paste hydration was higher than that of fly ash at this period. The SF had a significant effect on the plastic viscosity, yield stress, and fluidity of cement paste at this period [43]. 

## 5. Conclusions

This paper used the yield stress, plastic viscosity, flow spread, bleeding rate, and time-varying properties to characterize the macroscopic properties of the cement paste containing SF. The water film thickness theory, bleeding sedimentation model, microstructure analysis, and heat of hydration test methods were used to systematically explore the mechanism of SF on the macroscopic properties. The main conclusions obtained in this paper were as follows:

(1) The SF had different effects on the macro-fluidity and micro-rheological properties of cement paste under different w/c ratios. When the w/c ratio was 0.4, the addition of SF increased yield stress and plastic viscosity and decreased flow spread. The addition of SF increased the rheological parameters first and then decreased, and the corresponding fluidity first decreased and then increased at a w/c equal to 0.5 and 0.6. The effect of SF on rheological parameters was not significant, and the influence of SF on fluidity also had no obvious regularity when the w/c ratio was 0.7. The SF can effectively reduce the bleeding rate under different w/c ratios, which improved the stability of the cement paste.
(2) The time-varying properties of cement paste containing SF had the following regularity: the yield stress–time curve exhibited a linear increase, and the function form was *τ*(*t*) = *τ*_0_ + *k*_0_*t* and *τ*(*t*) = *τ*_1_ + *k*_1_*t*. The plastic viscosity–time curve exhibited an exponential function increase, and the function form can be described as *η*(*t*) = *η*_0_*e^at^*. The fluidity–time curve can be regarded as a function composed of a linear function and a quadratic function, and the specific form was *L*(*t*) = *L*_0_ − *k*_1_*t* and *L*(*t*) = *L*_0_ − *k*_1_*t* − *a*_1_*t*^2^. The SF made the rheological parameters increase slowly and fluidity attenuation degree decreased at the initial stage, but the effect was significantly enhanced after entering the accelerated hydration period.
(3) When the water–cement ratio was below a certain upper limit, WFT was an important factor affecting flow parameter and rheological parameters of cement paste. While WFT had no obvious correlation with flow parameter and rheological parameters at a w/c equal to 0.7.
(4) The SF can effectively reduce the bleeding rate of cement paste. This was due to the low density and small particle size of SF, whose sedimentation rate was less than that of fly ash particles. In addition, the incorporation of SF effectively alleviated the internal particle agglomeration and flocculation.
(5) An important reason for the time-varying changes of paste was that SF affected the hydration reaction. The hydration reaction of paste was slow during the induction period, and the rheological parameters and flow spread did not change significantly. After entering the acceleration period, due to the heterogeneous nucleation and dilution effect of SF, the hydration reaction was accelerated and more heat of hydration was released. The mass production of hydration products resulted in large changes in the rheology and fluidity of the paste.
(6) The application of SF as a new type of mineral admixture in the field of grouting was promising and meaningful. The substitution of SF can reduce the amount of cement while meeting the properties required for grouting, which can save economic costs and protect the environment. In the future, the combined use of SF and superplasticizer can be further explored in the field of grouting.

## Figures and Tables

**Table 1:** Chemical compositions of OPC, FA, and SF used in the experiment.

Phase	Mass Percentage (%)		
	OPC	FA	SF
SiO_2_	13.9	44.88	95.13
Al_2_O_3_	3.052	39.71	0
Fe_2_O_3_	4.801	3.69	0.71
CaO	72.374	5.48	2.16
MgO	1.075	0.60	0
Na_2_O	0.155	0.24	0.91
SO_3_	2.628	0.55	0.03
K_2_O	0.970	1.70	0.02
P_2_O_5_	0.228	0.52	0.14
TiO_2_	0.350	2.42	0
MnO	0.300	0.01	0
ZrO_2_	0.019	0.11	0.03
SrO	0.105	0.07	0.044
Cl	0.022	0.02	0.87

**Table 2:** Proportion of cement paste in the experiment.

Sample Code	Cement/%	Fly Ash/%	SF/%	w/c Ratio
A1	50	50	0	0.4/0.5/0.6/0.7
A2		40	10	
A3		30	20	
A4		20	30	
A5		10	40	
A6		0	50	

**Table 3:** Yield stress-time equations of cement paste containing SF.

w/c Ratio	Sample Code	Fitting Equation (First Period)	Fitting Equation (Second Period)
0.4	A1	τ=0.0186t+26.508	/
	A2	τ=0.0201t+26.955	/
	A3	τ=0.0201t+28.132	/
	A4	τ=0.0193t+28.741	/
	A5	τ=0.0196t+30.411	/
	A6	τ=0.0193t+31.172	/
0.5	A1	τ=0.0122t+14.385	τ=0.0373t+7.273
	A2	τ=0.0117t+15.035	τ=0.0413t+5.556
	A3	τ=0.0111t+14.017	τ=0.0461t+2.993
	A4	τ=0.0129t+15.534	τ=0.0403t+7.093
	A5	τ=0.0144t+16.971	τ=0.0465t+5.091
	A6	τ=0.0172t+17.544	τ=0.0472t+5.181
0.6	A1	τ=0.0119t+11.717	τ=0.0341t+5.063
	A2	τ=0.0108t+10.867	τ=0.0348t+4.075
	A3	τ=0.0104t+10.697	τ=0.0351t+3.391
	A4	τ=0.0131t+11.291	τ=0.0343t+5.282
	A5	τ=0.0145t+11.623	τ=0.0349t+6.095
	A6	τ=0.0169t+12.293	τ=0.0351t+7.147

**Table 4:** Plastic viscosity–time equations of cement paste containing SF.

w/c Ratio	Sample Code	Fitting Equation	R^2^
0.4	A1	$\eta =3.5249e^{0.0024t}$	0.968
	A2	$\eta =3.6781e^{0.0022t}$	0.9729
	A3	$\eta =3.872e^{0.0021t}$	0.9651
	A4	$\eta =3.9636e^{0.002t}$	0.948
	A5	$\eta =4.127e^{0.0019t}$	0.9863
	A6	$\eta =4.169e^{0.0019t}$	0.9649
0.5	A1	$\eta =0.418e^{0.008t}$	0.9048
	A2	$\eta =0.407e^{0.00934t}$	0.9386
	A3	$\eta =0.358e^{0.00982t}$	0.947
	A4	$\eta =0.423e^{0.00789t}$	0.9124
	A5	$\eta =0.224e^{0.00791t}$	0.9379
	A6	$\eta =0.336e^{0.00726t}$	0.9526
0.6	A1	$\eta =0.13e^{0.005t}$	0.9136
	A2	$\eta =0.12e^{0.0067t}$	0.945
	A3	$\eta =0.13e^{0.0086t}$	0.9322
	A4	$\eta =0.125e^{0.00929t}$	0.9848
	A5	$\eta =0.137e^{0.00824t}$	0.9542
	A6	$\eta =0.135e^{0.00979t}$	0.9858

**Table 5:** Flow spread–time equations of cement paste containing SF.

w/c Ratio	Sample Code	Fitting Equation (First Period)	Fitting Equation (Second Period)
0.4	A1	L=136.05−0.091t	/
	A2	L=133.82−0.0903t	/
	A3	L=131.75−0.0889t	/
	A4	L=126.61−0.0888t	/
	A5	L=125.46−0.0879t	/
	A6	L=112.2−0.0873t	/
0.5	A1	L=183.961−0.136t	$L=-0.0014t^{2}+0.613t+86.93$
	A2	L=188.867−0.125t	$L=-0.0017t^{2}+1.031t+26.091$
	A3	L=191.977−0.121t	$L=-0.0018t^{2}+0.726t+93.399$
	A4	L=180.161−0.116t	$L=-0.0021t^{2}+1.014t+29.417$
	A5	L=179.919−0.118t	$L=-0.0023t^{2}+0.923t+43.594$
	A6	L=172.514−0.113t	$L=-0.0024t^{2}+1.168t-2.753$
0.6	A1	L=237.044−0.0665t	$L=-0.0013t^{2}+0.718t+116.71$
	A2	L=239.065−0.0643t	$L=-0.0018t^{2}+0.9978t+81.48$
	A3	L=247.219−0.0613t	$L=-0.0017t^{2}+0.8123t+126.32$
	A4	L=231.993−0.0623t	$L=-0.0019t^{2}+0.6904t+160.91$
	A5	L=228.156−0.0605t	$L=-0.0021t^{2}+0.7991t+102.73$
	A6	L=223.811−0.568t	$L=-0.0022t^{2}+0.7463t+127.64$

## Data Availability

Data sharing is not applicable.
